# Sex-specific strategy use and global-local processing: a perspective toward integrating sex differences in cognition

**DOI:** 10.3389/fnins.2014.00425

**Published:** 2014-12-22

**Authors:** Belinda Pletzer

**Affiliations:** ^1^Department of Psychology, Paris-Lodron-University SalzburgSalzburg, Austria; ^2^Center for Cognitive Neuroscience, Paris-Lodron University SalzburgSalzburg, Austria

**Keywords:** sex differences, cognitive functions, Hemispheric asymmetries, sex hormones, cognitive strategies, global-local processing

## Abstract

This article reviews the literature on sex-specific strategy use in cognitive tasks with the aim to carve out a link between sex differences in different cognitive tasks. I conclude that male strategies are commonly holistic and oriented toward global stimulus aspects, while female strategies are commonly decomposed and oriented toward local stimulus aspects. Thus, the strategies observed in different tasks, may depend on sex differences in attentional focus and hence sex differences in global-local processing. I hypothesize that strategy use may be sex hormone dependent and hence subject to change over the menstrual cycle as evidenced by findings in global-local processing and emotional memory. Furthermore, I propose sex hormonal modulation of hemispheric asymmetries as one possible neural substrate for this theory, thereby building on older theories, emphasizing the importance of sex differences in brain lateralization. The ideas described in the current article represent a perspective toward a unifying approach to the study of sex differences in cognition and their neural correlates.

## He or she—short introduction to sex differences in cognition

For decades it has been debated, whether men and women differ in specific cognitive abilities (e.g., Halpern, 2000; Hyde, 2005; Andreano and Cahill, 2009). The present chapter shall by no means provide an extensive review of these sex differences, but rather introduce those sex differences, which are relevant for the idea presented in this article.

Sex-sensitive tasks have been identified in the domains of spatial, verbal, and memory performance. Thereby the largest sex differences, indicating a male superiority, have been reported for mental rotation tasks (e.g., Voyer and Bryden, 1990; Voyer et al., 1995; Schoning et al., 2007) with effect sizes ranging from 0.25 to 3.04 (Andreano and Cahill, 2009) and spatial navigation tasks (e.g., Galea and Kimura, 1993; Silverman et al., 2000; Saucier et al., 2002) with effect sizes ranging from 0.36 to 1.04 (Andreano and Cahill, 2009). In the domain of verbal abilities, sex differences have been reported for verbal fluency tasks (e.g., Capitani et al., 1998, 1999, 2005) with effect sizes ranging from 0.13 to 0.89 (Mann et al., 1990; Bolla et al., 1998; Loonstra and Sellers, 1998; Halari et al., 2005, 2006; De Frias et al., 2006; Gauthier et al., 2009; Hausmann et al., 2009; Soleman et al., 2013; Hirnstein et al., 2014), verbal memory tasks (e.g., Kimura and Seal, 2003; Yonker et al., 2003) with effect sizes ranging from 0.18 to 0.97 (Andreano and Cahill, 2009). But non-significant effects and effects to the opposite have also been reported (e.g., Kimura and Seal, 2003; Yonker et al., 2003; Munro et al., 2012). Sex differences have also been observed in autobiographic memory (Pillemer et al., 2003) and other aspects of episodic memory, like the recognition of odors (e.g., Oberg et al., 2002), faces (e.g., Bengner et al., 2006), objects, and pictures (e.g., Galea and Kimura, 1993), as well as for a variety of higher order cognitive functions (Harness et al., 2008; Li et al., 2009; Huster et al., 2011).

However, several of these findings have been questioned by theorists arguing that sex differences are overall small and negligible and within-group variation is stronger than the variation between groups (Hyde, 2006; Hyde and Linn, 2006). For example, a meta-analysis by Hyde and Linn (1988) yielded only a very weak effect size (*d* = 0.11) for sex differences in verbal functions across 165 studies using different verbal tasks. However, whether overall performance differences between men and women exist, does not explain why and how cognition differs between the sexes.

First, there is an increasing interest in the actions of sex hormones on the brain (De Frias et al., 2006), which may affect sex differences in cognitive tasks, emotional processing or personality. However, sex hormone levels, especially in women are not constant, but subject to changes due to endogenous hormonal fluctuations (menstrual cycle) or the application of synthetic steroids (hormonal contraception). Hence, for some tasks we do of course expect within-group variation in cognitive performance that may mask differences between groups, if these factors are not adequately controlled for (see also Pletzer et al., 2011, 2014a). It has for example been demonstrated that women perform better on mental rotation and other spatial tasks during the early follicular phase (low estrogen and progesterone) or if on hormonal contraception, while verbal abilities are also increased in hormonal contraceptive users as well as during the luteal cycle phase (high estrogen and progesterone) (Hampson, 1990; e.g., Mordecai et al., 2008; Wharton et al., 2008; Dadin et al., 2009). However, as outlined by a recent review (Sundstrom et al., this topic), these findings are not always replicable, also due to inconsistencies in the definition of cycle phases.

Second, it has been argued in the personality domain, that one cannot conclude from the comparison of isolated personality dimensions that differences between men and women in personality are small (Del Giudice et al., 2012). Rather personality has always been viewed as a multidimensional construct and research on sex differences should approach it as such. Personality dimensions do not affect our behavior individually. It is their relative manifestation and the interactions between different traits that form our behavior as a whole and these relations and interactions are what should be studied with respect to individual differences, as e.g., sex differences. I propose that the same holds true for the cognitive domain. While sex differences in some abilities may be small and sex differences are more pronounced in some abilities than others (e.g., spatial abilities), it is our cognitive profile, i.e., the common action and interaction of all aspects of cognition that shape our everyday life performance. Therefore, research on sex differences should advance from studying some cognitive abilities as separate entities, but move toward a more integrative approach and try to link sex differences across singular tasks. By identifying similarities between sex differences in various tasks, we may derive common principles and ideally link these principles to neural substrates.

Third, an absence of sex differences at the behavioral level does not necessarily imply that men and women did process a specific task in the same way. For example, several neuroimaging studies demonstrate sex differences in brain activation during a task, while not observing behavioral sex differences (e.g., Weiss et al., 2003; Schoning et al., 2007). Many authors have argued that men and women use different cognitive strategies (e.g., Cochran and Wheatley, 1989) employing different approaches. A common method is to utilize different instructions or the use of different stimulus materials or categories that favor the use of one strategy over another. For example, Sharps et al. (1993) were able to demonstrate that sex differences in a mental rotation task disappear, if they used non-spatial instructions and a female superiority in object location memory disappears, if the labeling of items is not possible (Postma et al., 2004). The use of different strategies may also be suggested by eye-tracking studies, if men and women focus on different stimulus aspects (e.g., Hampson, 1990). Another source of information regarding sex-specific strategy use are participants self-reports (e.g., Gluck, 2003). The following chapter will summarize several examples, where men and women approach a task with different strategies. As I will outline, it may be the nature of these differential strategies that links sex differences across tasks and to brain organization.

## Holistic or decomposed—dissociation of sex-specific strategies across tasks

The dissociation of cognitive strategies between men and women has been studied most extensively in spatial tasks. In the mental rotation task for example, it has been deduced from participants self-reports that men tend to use a more holistic “Gestalt” approach, while women use a segmentary strategy of rotating parts of the stimuli separately (Gluck, 2003; Pena et al., 2008; Rilea, 2008). In spatial navigation, it has been demonstrated using participants self-reports and different instructions, that men focus on distal landmarks and use allocentric coordinates, while women focus on local landmarks and use egocentric coordinates (Galea and Kimura, 1993; Lawton, 1994, 2001; Lawton et al., 1996). Men outperform women in both real world and 2D-matrix navigation, when directions are given in Euclidean terms using allocentric coordinates (Saucier et al., 2002). On the other hand, women outperform men, when directions are given using landmark information and egocentric coordinates (Saucier et al., 2002) and sex differences in virtual navigation decline the more landmark information is available (Andersen et al., 2012). For navigation, the sex-specific strategy dissociation has been corroborated by eye-tracking evidence. In a virtual water maze, men explore more space, while women show longer fixation durations (Mueller et al., 2008). Furthermore, the allocentric strategy has successfully been related to mental rotation performance (Saucier et al., 2002), demonstrating that a more global strategy is beneficial in spatial tasks.

A dissociation between global and local strategies has however also been described for a variety of spatial-related and non-spatial tasks. For example, using different stimulus categories, we recently described a strategy dissociation in a number comparison task (Pletzer et al., 2013), suggesting that men process multi-digit numbers in a more holistic fashion (whole numbers), while women process decade and unit digit magnitudes separately.

Likewise, eye-tracking evidence demonstrated that during face and emotion recognition women fixate more strongly on the eyes, independent of view-point, while men tend to focus their gaze more toward the nose in frontal views and the cheeks in profiles, i.e., the view-specific center-of-gravity (e.g., Saether et al., 2009).

The common denominator across the tasks and strategies described so far, is that the sex specific strategies can be linked to visuospatial attention in that participants either self-report their focus of attention, their focus of attention is actively directed toward particular stimulus aspects via different instructions or the use of different stimulus categories, or their focus of attention is recorded using eye-tracking evidence.

Thereby, male strategies appear to share the common feature of being oriented toward more global stimulus features or aspects of the task, i.e., they can be described as holistic. Female strategies however appear to be oriented toward more local stimulus features and can thus be described as decomposed. Consequently, sex-specific strategy use may be linked to sex differences in attentional focus, an idea which will be pursued in the next chapter.

The global-local dissociation has been studied particularly well in the context of emotional memory. It has repeatedly been demonstrated that men show better memory of the gist of an emotional story, while women better remember the details of an emotional story (e.g., Cahill, 2003; Cahill et al., 2004; Nielsen et al., 2011). Thereby, the gist and the detail refer to aspects of visual scenes, which makes it plausible that the strategies described for emotional memory are also linked to differences in visuo-spatial attention.

It is an interesting question, whether sex-specific strategies in other tasks that cannot as easily be related to visuo-spatial attention, can also be linked to this principle. It has for example repeatedly been demonstrated that during verbal fluency tasks, men produce larger clusters (series of words that belong to the same semantic or phonological category), while women tend to switch more often between different categories (Weiss et al., 2006). Category size has been used as one indicator of conceptual global-local processing, as described in the next chapter (Darwent et al., 2010).

In summary, sex-specific strategy use has been reported for almost every cognitive task for which sex differences in performance have been reported. For several tasks, these strategies may be generalizable via a principle in visuo-spatial attention with male strategies being oriented toward global stimulus aspects and female strategies being oriented toward local stimulus aspects. Note however, that only few of the studies described above (Nielsen et al., 2011, 2013; Pletzer et al., 2013) controlled for menstrual cycle phase, hormonal contraceptive use or sex hormone levels. In emotional memory for example, the focus on the details of an emotional story in women appears to be particularly enhanced during the luteal cycle phase, when women's estradiol and progesterone levels are high (Nielsen et al., 2011, 2013). Thus, for several of these tasks, a hormonal modulation of sex-specific strategy use remains yet to be established.

## Global or local—sex differences in attentional focus

Individual differences in global-local processing are well-established (Forster and Dannenberg, 2010a,b). Perceptual global-local processing refers to the tendency to process visual stimuli as a whole or in parts, whereas conceptual global-local processing refers to the tendency to think in more concrete or abstract terms (Darwent et al., 2010).

Perceptual global-local processing is traditionally studied using hierarchical stimuli (Navon paradigm), i.e., a global structure made up of local parts (Navon, 1977). These stimuli allow assessing global and local processing independently of each other and in their interaction, since participants attention can be directed toward either the global or the local level. Thereby, participants are asked to respond if a certain predefined target appears at a specified level. From experiments with this paradigm, the concept of global precedence was developed (Navon, 1977, 1981), which includes among others the observation that reactions to global targets are faster than reactions to local targets (***global advantage***).

If sex differences in cognitive tasks are based on sex differences in attentional focus, these differences should be apparent in a Navon paradigm. Indeed several findings have been published that support this view. Using a divided attention paradigm, a local advantage has been demonstrated in women that was absent in men (Roalf et al., 2006), while in a selective attention paradigm, a global advantage has been demonstrated in men that was absent in women (Razumnikova and Vol'f, 2011). However, these findings have been questioned by a lack of sex differences in global advantage using hierarchical line/shape stimuli (Kimchi et al., 2009) or a similarity judgment task (Basso and Lowery, 2004).

In a recent study designed to resolve inconsistencies between these previous reports, we demonstrated that the reduced global advantage in women is strongly dependent on hormonal status (Pletzer et al., 2014b). Women in their luteal phase (high estradiol and progesterone) showed reduced global advantage in comparison to men, but also in comparison to women in their follicular phase (low estradiol and progesterone) and hormonal contraceptive users. Furthermore, global advantage was positively related to testosterone levels, but negatively to progesterone levels, while no relationship was observed with estradiol levels. Thus, an enhanced focus on the global aspects of hierarchical stimuli is probably facilitated by testosterone, while an enhanced focus on the local aspects of hierarchical stimuli is probably facilitated by progesterone. This may explain why only women in their luteal cycle phase, i.e., women with elevated progesterone levels, showed a reduction in global advantage compared to men.

Conceptual global-local processing is traditionally studied via construal level tasks (Darwent et al., 2010), e.g., asking participants to group a given set of words into categories, whereby category size is used as an indicator of global-local processing. To the best of our knowledge, sex differences have not been as explicitly studied, nor consistently been reported for this task, although certain similarities to the verbal fluency or verbal memory tasks are apparent. However, a link between perceptual and conceptual global-local processing has been proposed (Forster, 2009; Darwent et al., 2010; Forster and Dannenberg, 2010a,b). Thus, it may be worth investigating a possible link of sex differences in strategy selection between visuo-spatial and verbal tasks. There is for example evidence for sex differences in the relation between creativity and perceptual global-local processing (Razumnikova and Vol'f, 2012).

Furthermore, several psychological (e.g., mood) or social (e.g., stereotype threat) factors have been identified that can affect the size of sex differences in cognitive tasks (e.g., Hausmann et al., 2009; Hirnstein et al., 2014). Global-local processing has been linked to mood (Basso et al., 1996) and gender stereotype activation (Anderson, 2011), which suggests that these factors should also been taken into account when establishing a link between sex-specific strategy use and global-local processing across different tasks.

## Left or right/coupling or decoupling—hemispheric interplay as neural substrate of sex differences?

Results from visual hemifield (e.g., Robertson and Lamb, 1991), EEG (e.g., Johannes et al., 1996) and fMRI studies (Fink et al., 1996) indicate, that the right hemisphere shows an advantage for processing of the global level, while the left hemisphere shows an advantage for processing of the local level.

Hemispheric asymmetries have also been assessed for several of the cognitive functions discussed above as being subject to sex-specific strategy use (for reviews see e.g., Wada, 2009; Renteria, 2012). For example, verbal functions appear to be left-lateralized, while visuospatial functions appear to be right-lateralized. Such a lateralization of brain functions is mostly assumed to rely on inter-hemispheric inhibition (Chiarello and Maxfield, 1996), with the hemisphere dominant for a task inhibiting the non-dominant hemisphere. Since a right-hemispheric dominance for visuo-spatial attention has been reported (Heilman and Van Den Abell, 1980; Heilman et al., 1983), the lateralization of global-local processing may explain the general observation of a global advantage. The dominant right hemisphere is responsible for global processing and inhibits the non-dominant left hemisphere responsible for local processing.

Furthermore, sex differences in brain lateralization have received much attention. Some authors argue that hemispheric asymmetries are more pronounced in men than in women and that a stronger variation in hemispheric asymmetries is apparent in women as compared to men (for reviews see McGlone, 1980; Hausmann and Bayer, 2010; Renteria, 2012). However, a meta-analysis by Voyer (Voyer et al., 2012) suggests that this idea may not hold across tasks and that sex differences in the lateralization of verbal and visuo-spatial functions are modulated by modality (visual vs. auditory). Hausmann and Bayer (2010) argue that sex differences in hemispheric asymmetries may be modulated by intra- and inter-individual variations in sex hormone levels. A menstrual cycle dependent modulation of lateralization may explain the stronger variation in hemispheric asymmetries within the female group (Hausmann and Bayer, 2010).

In that respect it has been demonstrated that the lateralization of brain functions is particularly reduced during the luteal cycle phase, when a woman's estradiol and progesterone levels are high (Hausmann and Gunturkun, 2000). This reduction in hemispheric lateralization has originally been attributed to a progesterone-mediated reduction in inter-hemispheric inhibition, termed ***progesterone-mediated inter-hemispheric decoupling*** (Hausmann and Gunturkun, 2000). Therefore, we hypothesized that the reduction in global advantage we observed during the luteal cycle phase (Pletzer et al., 2014b), was mediated via the progesterone-dependent reduction of inter-hemispheric inhibition during global-local processing. However, recent studies stress the role of estradiol in modulating inter-hemispheric communication (Hausmann and Gunturkun, 2000; Weis et al., 2008; Weis and Hausmann, 2010; Hausmann et al., 2013) over the menstrual cycle and this theory has recently been extended to include sex hormone modulations of inter-hemispheric excitation and integration (Bayer et al., 2008). A relationship between global advantage and estradiol was not observed in our previous study (Pletzer et al., 2014b). A reduction of inter-hemispheric inhibition during high-hormone phases has been demonstrated in visual hemifield (Hausmann and Gunturkun, 2000), and fMRI experiments (Weis et al., 2008). In line with this idea, sex-specific hemispheric specialization has recently been demonstrated during global-local processing in a Navon paradigm (Lee et al., 2012), as well as during emotional memory (Cahill, 2007).

Other theories stress, that some sex hormones may enhance the functioning and intra-hemispheric integration of a particular hemisphere (e.g., Hampson, 1990). It has e.g., been proposed that the “female” sex hormone estrogen enhances left-hemisphere functioning, while the “male” sex hormone testosterone has been discussed to enhance right-hemisphere functioning (e.g., Toga and Thompson, 2003). The latter view is in line with our observation that high levels of testosterone are associated with enhanced global advantage.

While a complete picture of how sex hormones interact with inter-hemispheric communication has yet to emerge, several results indicate that sex hormones modulate inter-hemispheric communication (see Hausmann and Bayer, 2010 for a review). We hypothesize that via this modulation, sex hormones affect global and local attention, which may relate to cognitive strategies in several cognitive tasks. To establish a more complete picture on the sex hormonal modulation of lateralization and the link between lateralization and cognitive strategies, hemispheric asymmetries should be taken into account when studying sex differences in cognitive strategies.

## Conclusions

In summary, it has been found, that sex specific strategies share several features over different cognitive tasks and can be described as global/holistic in men and local/decomposed in women and linked to sex-differences in global-local processing. I hypothesized that sex differences in the lateralization of brain functions accompany these strategies as a result of sex hormone modulation of transcallosal neurotransmission. Empirical evidence linking strategy to lateralization and demonstrating hormone-dependent modulation of strategies as well as lateralization is still lacking for several of the tasks described. This shifts the emphasis from a descriptive comparison of men and women to the question how sex hormones modulate cognition as a whole and can only be answered by an adequate understanding of the changes occurring over the course of the menstrual cycle. The idea of the corpus callosum, gating attentional focus and thereby guiding strategy use in a variety of cognitive tasks represents a perspective toward a link between sex differences in different cognitive tasks.

### Conflict of interest statement

The author declares that the research was conducted in the absence of any commercial or financial relationships that could be construed as a potential conflict of interest.
